# Comparative analysis of septic injury-inducible genes in phylogenetically distant model organisms of regeneration and stem cell research, the planarian *Schmidtea mediterranea *and the cnidarian *Hydra vulgaris*

**DOI:** 10.1186/1742-9994-5-6

**Published:** 2008-04-27

**Authors:** Boran Altincicek, Andreas Vilcinskas

**Affiliations:** 1 Interdisciplinary Research Center, Institute of Phytopathology and Applied Zoology, Justus-Liebig-University of Giessen, Heinrich-Buff-Ring 26-32, D-35392 Giessen, Germany

## Abstract

**Background:**

The planarian *Schmidtea mediterranea *and the cnidarian *Hydra vulgaris *have emerged as valuable model organisms in regeneration and stem cell research because of their prominent ability to regenerate a complete organism from any small body fragment. Under natural conditions wounding may result from predator attacks. These injuries open their innermost to a wide array of microbes present in the environment. Therefore, we established the hypothesis that regeneration processes may be linked to or at least accompanied by innate immune responses. In order to screen for septic wounding inducible genes we dissected individuals using a scalpel in the presence of a crude bacterial lipopolysaccharide preparation that is commonly used to elicit innate immune responses in animals and applied the suppression subtractive hybridization technique that selectively amplifies cDNAs of differentially expressed genes.

**Results:**

This analysis revealed the induced expression of 27 genes in immune challenged *Schmidtea *and 35 genes in immune challenged *Hydra*. Identified genes from both animals encode proteins that share sequence similarities with potential homologues from other organisms known to be involved in signaling (e.g. calreticulin in *Schmidtea *and major vault protein in *Hydra*), stress responses (e.g. Hsp20 in *Schmidtea *and a PRP19/PSO4 DNA repair protein in *Hydra*), or to represent potential antimicrobial effectors (e.g. perforin-like protein in *Schmidtea *and PR-1-like protein and neutrophil cytosolic factor 1 in *Hydra*). As expected, septic wounding also induces expression of genes in *Schmidtea *and *Hydra *potentially involved in tissue remodeling associated with regeneration processes (e.g. matrix metalloproteinase in *Schmidtea *and a potential von Willebrand factor in *Hydra*).

**Conclusion:**

We identified numerous immune-inducible genes in *Hydra *and *Schmidtea *that show a similar distribution corresponding to their physiological roles, although lineages of both animals split from their common ancestor for more than five hundred millions of years. The present study is the first analysis of immune-inducible genes of these two phylogenetically distant model organisms of regeneration and provide numerous candidate genes that we can use as a starting point for comparative examination of interrelationships between immunity and homeostasis.

## Background

To date, there is no satisfactory answer to the question why some animals have higher regeneration capacities than others. The ability to replace lost or injured body parts is widely distributed among animals, whereas regeneration of a complete organism from any small body fragment is restricted to only few animal phyla and is accompanied by the ability to reproduce asexually by budding or fission [1,2]. These features have been attributed to a stable population of stem cells known as neoblasts in *Schmidtea mediterranea *[3,4] and to both stem cell-based mechanisms and transdifferentiation in *Hydra vulgaris *[5,6]. These two phylogenetically distant animals with remarkable regeneration capacities attract renewed attention as powerful model organisms since both, *S. mediterranea *and *H. vulgaris*, are amenable to systemic RNAi mediated gene silencing and other genetic tools for functional gene analyses [7-11].

**Figure 1:**
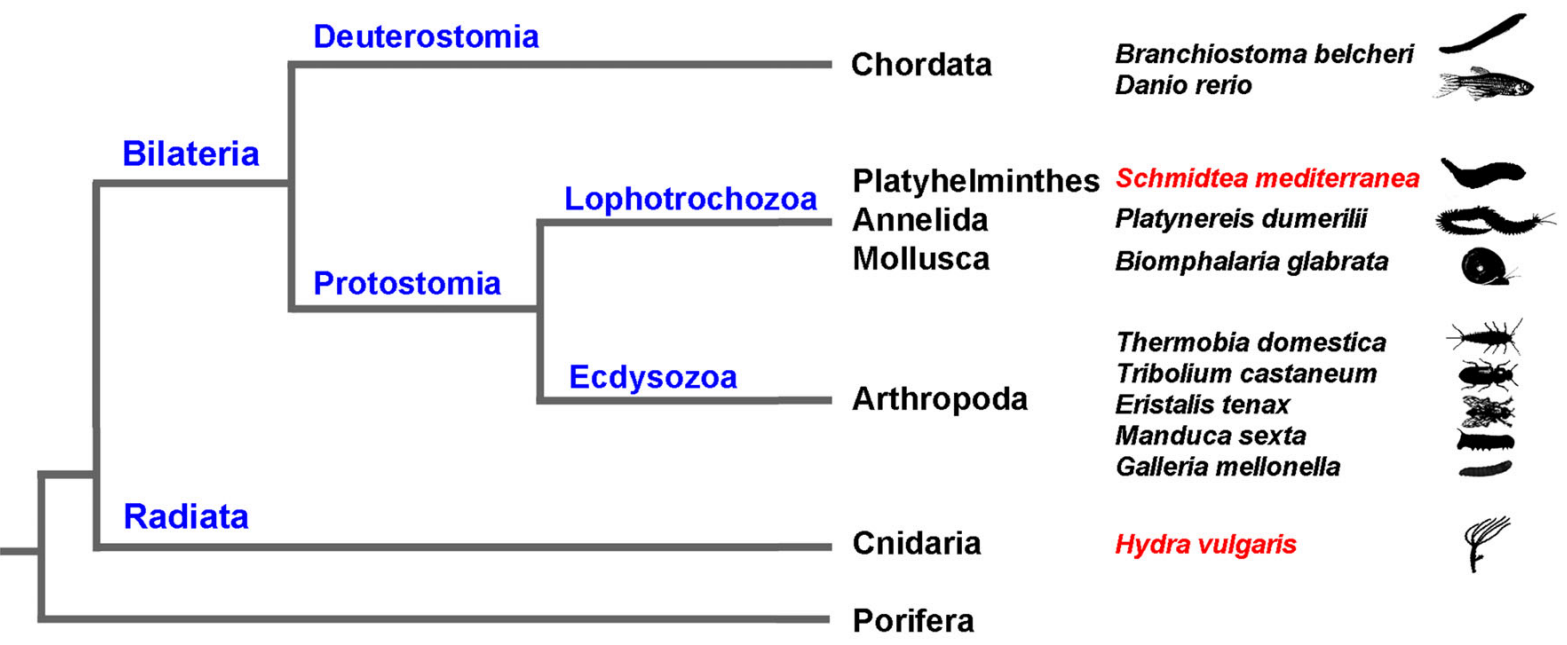
**Phyla distribution of animals whose immune-inducible genes have been analyzed by the SSH method**. A major evolutionary divide occurs in the animal kingdom between the so-called radially symmetric animals, which includes the cnidarian *Hydra*, and the bilaterally symmetric animals, which includes *Schmidtea*. The phylogenetic position of these two model organisms that have been selected for investigations in the present work (indicated by red color) promotes comparative analysis that may help to reconstruct functions and evolution of genes involved in regeneration and immunity. Previously, *Branchiostoma belcheri *[23], *Danio rerio *[24], *Platynereis dumerilii *[19], *Biomphalaria glabrata *[20,21], *Thermobia domestica *[12], *Tribolium castaneum *[14], *Eristalis tenax *[13], *Manduca sexta *[17], and *Galleria mellonella *[15] have been analyzed by the SSH method.

In their habitats *Hydra *and *Schmidtea *may be wounded by attacks from predators. These natural injuries open their innermost to a wide array of microbes present in the environment. Therefore, we established the hypothesis that regeneration processes may be linked to or at least accompanied by innate immune responses. As a first step towards understanding the immune defense reactions of both model organisms we used the suppression subtractive hybridization (SSH) technique. This method has been proven as a valuable tool for identification of novel immune-inducible genes in a number of animal species, including representatives of Ecdysozoa [12-18], Lophotrochozoa [19-22], and Deuterostomia [23,24]. Here, we applied the SSH method to identify genes that are differentially expressed upon wounding in Cnidaria and Platyhelminthes. We selected *Hydra *and *Schmidtea *for analyses because both are currently emerging as genetically tractable models in regeneration, development and stem cell research. In addition, their complete genome sequences have recently been determined and will be available soon [25,26]. The phylogenetic relationship between *Hydra *and *Schmidtea *and model organisms whose septic-injury inducible genes have previously been identified using the SSH-method supports comparative approaches to reconstruct evolution and function of genes that are linked to immunity (Fig. 1). Further reasons that make *Hydra *and *Schmidtea *amenable to comparative approach analyses are that both share similar body sizes and colonize freshwater habitats allowing comparable experimental conditions.

In this study, we report profiles of injury-inducible genes from *Schmidtea *and *Hydra*, including genes that encode proteins potentially involved in e.g. immune-inducible signaling and defense reactions. Comparisons of potential calreticulin, matrix metalloproteinase (MMP), and perforin with homologues from other organisms provide novel insights into the ancestral complexity and evolution of the metazoan immune system.

## Results and Discussion

### Subtracted cDNA libraries of immune challenged *S. mediterranea *and *H. vulgaris*

**Figure 2:**
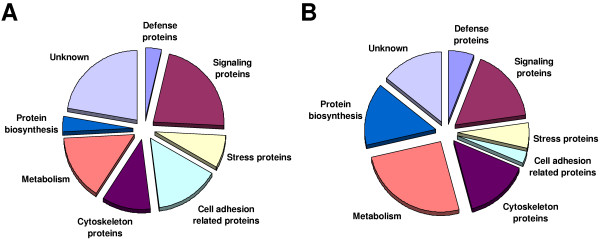
**Distribution of immune-inducible transcripts identified by the SSH approach in *Schmidtea *and *Hydra***. Septic injury results in an immune-induced expression of totally 27 genes in *S. mediterranea ***(A) **and 35 genes in *H. vulgaris ***(B) **as obtained by the SSH approach. Interestingly, the genes have a similar distribution regarding their physiological roles.

A subtracted cDNA library enriched in immune-inducible genes from *S. mediterranea *and *H. vulgaris*, respectively, was constructed by using the SSH method. A total of each 288 clones were randomly picked and subjected to colony PCR. Plasmids of bacterial colonies that have been screened positively in blot hybridization indicating induced expression of corresponding genes were isolated and sequenced. Here, we describe the identification of 27 septic wounding-inducible genes in *S. mediterranea *(Table 1) and 35 septic wounding-inducible genes in *H. vulgaris *(Table 2) potentially involved in antimicrobial defense, signaling, and other immunity-linked cellular processes (Fig. 2).

**Table 1:** cDNAs from the subtracted *S. mediterranea *library.

Cluster	GenBank Accession No. of EST	Highest BlastX match	*E *value	PFAM/InterPro
*Defense protein*				
	FC823226	AAR82935: macrophage expressed protein (*Haliotis corrugata*)	3e-22	Membrane attack complex component/perforin/C9
*Signaling proteins*				
	FC823227	XP_001119958: similar to p21/Cdc42/Rac1-activated kinase 1 (*Apis mellifera*)	2e-35	P21-activated kinase
	FC823228	XP_972570: similar to PhosphoLipase C family member (plc-1) (*Tribolium castaneum*)	8e-09	
	FC823229	NP_524378: sarah CG6072-PA (*Drosophila melanogaster*)	5e-23	Calcipressin
	FC823230	CAK04334: similar to human chromodomain helicase DNA binding protein 2 (CHD2) (*Danio rerio*)	6e-53	DEAD-like helicases, N-terminal
	FC823231	XP_001197550: similar to mFLJ00175 protein, partial (*Strongylocentrotus purpuratus*)	2e-72	C2 calcium-dependent membrane targeting
	FC823232	XP_392689: similar to Calreticulin CG9429-PA (*Apis mellifera*)	8e-99	Calreticulin
*Stress related proteins*				
	FC823233	AAW25328: SJCHGC09367 protein (*Schistosoma japonicum*)	7e-07	Hsp20/alpha crystallin family
	FC823234	XP_696859: hypothetical protein (*Danio rerio*)	7e-27	RAD50
*Cell adhesion related proteins*				
	FC823235	CAA37667: pre-pro-hatching enzyme (*Paracentrotus lividus*)	4e-20	Peptidase_M10
	FC823236	AAX25986: SJCHGC09378 protein (*Schistosoma japonicum*)	3e-52	Collagen triple helix repeat
	FC823237	ZP_01611880: putative adhesion lipoprotein (*Alteromonadales bacterium TW-7*)	5e-02	Beta-Ig-H3/fasciclin
	FC823238	AAX25800: SJCHGC02149 protein (*Schistosoma japonicum*)	4e-06	Cell adhesion molecule
*Cytoskeleton proteins*				
	FC823239	XP_419249: hypothetical protein (*Gallus gallus*)	2e-64	Tubulin/FtsZ
	FC823240	XP_001176242: similar to cytoskeletal actin CyIIb (*Strongylocentrotus purpuratus*)	3e-38	Actin
	FC823241	XP_624678: similar to Myosin IA (MIA) (Brush border myosin IA) (BBMIA) (*Apis mellifera*)	8e-61	IQ calmodulin-binding region; Myosin_head
*Metabolism*				
	FC823242	AAB53748: 3-hydroxy-3-methylglutaryl-CoA reductase (*Oryza sativa*)	1e-14	Hydroxymethylglutaryl-coenzyme A reductase
	FC823243	CAF95654: unnamed protein product (*Tetraodon nigroviridis*)	2e-21	Selenophosphate synthetase
	FC823244	AAU03135: glutamate dehydrogenase (*Hylobates lar*)	1e-55	Glu/Leu/Phe/Val dehydrogenase
	FC823245	XP_001177631: similar to Gldc-prov protein (*Strongylocentrotus purpuratus*)	4e-52	Glycine cleavage system P-protein
*Protein biosynthesis*				
	FC823246	AAL49199: RE63456p (*Drosophila melanogaster*)	9e-18	Ribosomal_L34e
*Unknown proteins*				
	FC823247	CAE65027: Hypothetical protein CBG09865 (*Caenorhabditis briggsae*)	1e-03	
	FC823248	Hypothetical protein	NSM^1^	Ubiquitin
	FC823249	Hypothetical protein	NSM	
	FC823250	Hypothetical protein	NSM	
	FC823251	Hypothetical protein	NSM	
	FC823252	Hypothetical protein	NSM	

^1^NSM, no significant match

**Table 2:** cDNAs from the subtracted *H. vulgaris *library.

Cluster	GenBank Accession No. of EST	Highest BlastX match	*E *value	PFAM/InterPro
*Defense proteins*				
	FC823190	XP_001356627: GA14264-PA (*Drosophila pseudoobscura*)	5e-07	SCP/Tpx-1/Ag5/PR-1/Sc7 family of extracellular domains
	FC823191	XP_001063612: similar to neutrophil cytosolic factor 1 (*Rattus norvegicus*)	7e-35	p47-phox (neutrophil cytosolic factor 1)
*Signaling proteins*				
	FC823192	XP_690149: similar to diacylglycerol kinase eta2 (*Danio rerio*)	4e-18	
	FC823193	XP_001201815: hypothetical protein, partial (*Strongylocentrotus purpuratus*)	6e-96	Major vault protein, N-terminal
	FC823194	XP_001489489: similar to THO complex 4 (*Equus caballus*)	6e-06	
	FC823195	AAZ99727: dickkopf-like protein Dlp-1 precursor (*Hydra magnipapillata*)	4e-44	
	FC823197	XP_421629: similar to Transmembrane 9 superfamily protein member 3 precursor (*Gallus gallus*)	2e-79	Nonaspanin (TM9SF)
	FC823198	Q6NZZ4: Transmembrane protein 107 (*Danio rerio*)	3e-21	
*Stress related proteins*				
	FC823199	XP_867913: similar to PRP19/PSO4 homologue (*Canis familiaris*)	1e-91	WD40/YVTN repeat-like
	FC823200	AAM76192: LD36241p (Scythe protein) (*Drosophila melanogaster*)	5e-03	
*Cell adhesion related proteins*				
	FC823201	AAT37632: von Willebrand factor (*Branchiostoma belcheri tsingtaunese*)	4e-11	
*Cytoskeleton proteins*				
	FC823202	EAW96898: myosin, light polypeptide 6B, (*Homo sapiens*)	1e-10	Calcium-binding EF-hand
	FC823203	CAA48796: actin (*Podocoryne carnea*)	3e-116	Actin/actin-like
	FC823204	P18320: Profilin (*Anthocidaris crassispina*)	5e-41	Profilin/allergen
	FC823205	P34032: Thymosin beta-4 (*Oryctolagus cuniculus*)	6e-05	Thymosin beta-4
	FC823206	CAM37732: flagellar calcium-binding protein, putative (*Leishmania braziliensis*)	7e-16	Calcium-binding EF-hand
*Metabolism*				
	FC823207	XP_001366187: similar to NADH dehydrogenase (*Monodelphis domestica*)	6e-02	NADH dehydrogenase
	FC823208	XP_395823: similar to Phosphomannomutase (*Apis mellifera*)	1e-25	Phosphomannomutase
	FC823209	XP_001197762: similar to NAD(P)H:quinone oxidoreductase (*Strongylocentrotus purpuratus*)	1e-15	Oxidoreductase
	FC823210	XP_001494492: hypothetical protein (*Equus caballus*)	3e-15	MoeA, N-terminal, domain I and II
	FC823211	XP_783197 hypothetical protein (*Strongylocentrotus purpuratus*)	3e-24	Methyltransferase type 11
	FC823212	NP_001017016: exosome component 6 (*Xenopus tropicalis*)	8e-05	Exonuclease, RNase T and DNA polymerase III
	FC823213	NP_957038: small fragment nuclease (*Danio rerio*)	3e-50	Ribonuclease PH related
	FC823214	NP_990451: enolase 1 (*Gallus gallus*)	3e-99	Enolase
	FC823215	ABC25045: mitochondrial succinate dehydrogenase flavoprotein subunit (*Hydra vulgaris*)	4e-94	Fumarate reductase/succinate dehydrogenase flavoprotein,
*Protein biosynthesis*				
	FC823216	AAR09792: similar to Drosophila melanogaster eIF-5A (*Drosophila yakuba*)	2e-06	eIF-5A
	FC823217	NP_001016714: ribosomal protein L28 (*Xenopus tropicalis*)	8e-15	Ribosomal protein L28e
	FC823218	AAY43411: ribosomal protein L23 (*Phytophthora infestans*)	4e-34	Ribosomal protein L14b/L23e
	FC823219	XP_392565: similar to Ribosomal protein L18 (*Apis mellifera*)	4e-60	Ribosomal protein L18e
	FC823220	AAY97868: cytoplasmic ribosomal protein S13 (*Lycopersicon esculentum*)	1e-47	Ribosomal protein S15
*Unknown proteins*				
	FC823221	XP_413837: hypothetical protein (*Gallus gallus*)	6e-02	
	FC823222	ZP_01629577: hypothetical protein (*Nodularia spumigena CCY9414*)	9e-02	
	FC823223	Hypothetical protein	NSM^1^	
	FC823224	Hypothetical protein	NSM	
	FC823225	Hypothetical protein	NSM	

^1^NSM, no significant match

### Signaling

In animals, hereditable receptors including the prominent Toll-receptors recognize damage- or pathogen-associated molecular pattern molecules and engage multiple immune-related signaling pathways [27]. Here, we identified a *Schmidtea *cDNA encoding a protein that exhibits highest sequence similarities to p21/Cdc42/Rac1-activated kinase 1 from *Apis*. In mammals, this kinase is believed to act directly on the JNK (c-Jun N-terminal kinase) MAP (mitogen-activated protein) kinase pathway [28]. JNK is a prominent stress kinase that has been studied mostly in the context of cellular stress and apoptotic cell death following, for example, heat shock, DNA damage, and inflammation [29].

Calcium signals in human immune cells participate in the regulation of cell differentiation and influence lymphocyte motility, immunological synapse formation, degranulation and phagocytosis [30]. In agreement, we found several predicted proteins in *Schmidtea *and *Hydra *that show similarities to members of the calcium signaling pathways suggesting that calcium pathways may be important in immune responses in these animals. One *Schmidtea *protein with highest similarities to ferlin family proteins that are known to be associated with both plasma and nuclear membranes contains a C2 domain that may play a role in calcium-mediated membrane fusion events during membrane regeneration and repair [31]. Additionally, we identified a *Schmidtea *cDNA that encodes a potential calcipressin homologue. Vertebrate calcipressins modulate the pattern of calcineurin-dependent transcription, and may influence calcineurin activity beyond calcium to integrate a broad array of signals into the cellular response [32]. The importance of calcineurin in immunity is highlighted by the use of calcineurin inhibitors such as cyclosporine as prominent immunosuppressive drugs in humans [33]. Furthermore, we found a potential *Schmidtea *phospholipase C that may generate inositol triphosphate and diacylglycerol by hydrolyzing phosphatidylinositol which in turn leads to raising the level of intracellular calcium. In *Hydra*, we determined the induced expression of a potential diacylglycerol kinase that balances between two signaling lipids, diacylglycerol and phosphatidic acid, by phosphorylating the former one. Therefore, diacylglycerol kinases may be involved in numerous lipid and calcium signaling pathways on transient or continuous demands, respectively [34].

**Figure 3:**
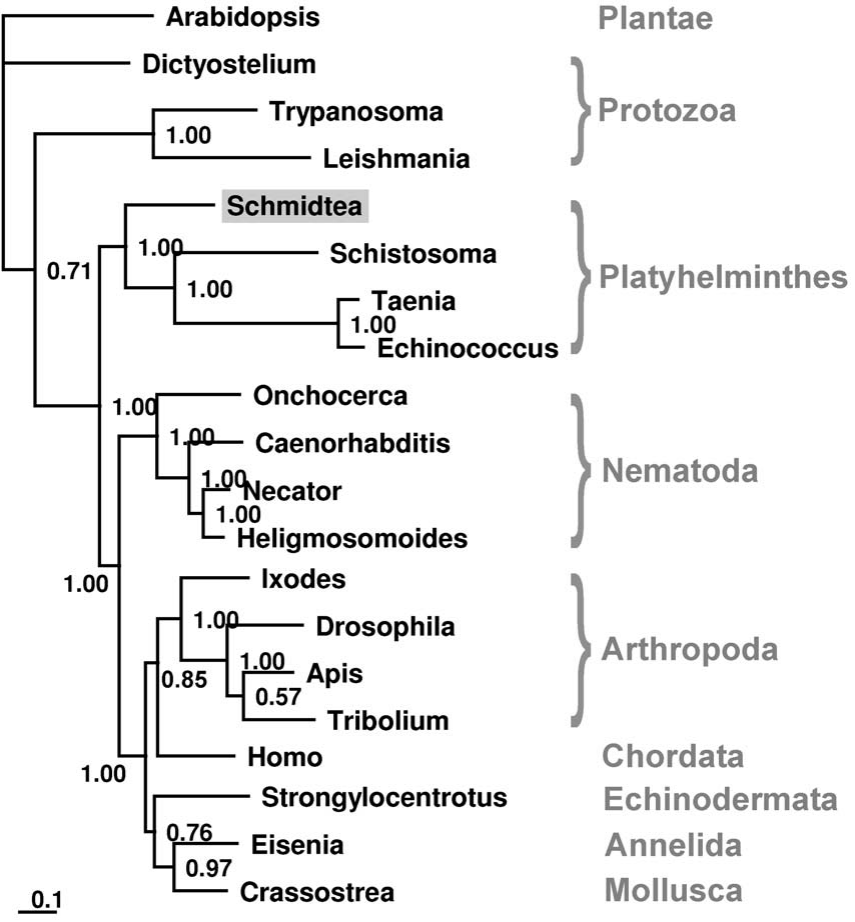
**Phylogenetic analysis of *Schmidtea *calreticulin and homologues from selected species**. We identified a calreticulin protein in the *Schmidtea *cDNA library. Using corresponding EST sequence and further EST sequences from the public database (DN316126 and EG417940) we constructed the potential full-length protein. A Bayesian protein tree was generated using calreticulin sequences from *Schmidtea *and from selected species (*Trypanosoma cruzi*, EAN82340; *Leishmania infantum*, XP_001467584; *Dictyostelium discoideum*, XP_639010; *Eisenia fetida*, ABI74618; *Schistosoma japonicum*, ABK34456; *Taenia solium*, AAK52725; *Echinococcus granulosus*, AAX73173; *Apis mellifera*, XP_392689; *Drosophila melanogaster*, NP_524293; *Tribolium castaneum*, XP_971763; *Crassostrea gigas*, BAF63639; *Onchocerca volvulus*, AAA59056; *Necator americanus*, CAA07254; *Heligmosomoides polygyrus*, CAL30086; *Caenorhabditis elegans*, NP_504575; *Ixodes scapularis*, AAT99573; *Homo sapiens*, NP_004334; *Strongylocentrotus purpuratus*, NP_999643). As outgroup we selected plant *Arabidopsis thaliana *calreticulin (NP_001031199). This phylogenetic analysis reveal that calreticulins are evolutionarily conserved and that *Schmidtea *calreticulin (indicated by gray shading) form an own group with calreticulins from parasitic Platyhelminthes. The scale bar represents the substitutions per site.

In line with these findings, we identified a *Schmidtea *calreticulin homologue which is evolutionarily conserved (Fig. 3). Although originally characterized as a regulator of calcium homeostasis, more recently it has been shown to be present on the surface of many mammalian cell types and has been implicated in signal transduction events associated with innate immunity, cell adhesion, angiogenesis, and apoptosis [35]. In regard to its immune functions, cell surface levels of calreticulin directly correlate with the ability of human dentritic cells and polymorphonuclear phagocytes to mediate phagocytosis, and the capacity of normal and tumor cells to adhere to components of the extracellular matrix [35]. Furthermore, calreticulin has recently been demonstrated to play a crucial role in the loading of MHC class I molecules with optimal peptide cargo in mammals [36]. This is a clear example of ancient mechanisms co-opted by new ones such as the acquired immunity of vertebrates in the context of the evolution of the immune system. However, calreticulin is also a prominent stress-inducible molecular chaperone in the endoplasmatic reticulum. This is in agreement to our further identification of stress-related proteins, including a potential Hsp20/α-crystallin protein and a RAD50 DNA repair protein from *Schmidtea *and a PRP19/PSO4 DNA repair protein and a scythe protein (also known as Bat3) in *Hydra*. The latter one is an important regulator of the apoptogenic mitochondrial intermembrane protein AIF (Apoptosis-Inducing Factor) in mammals [37] and may have similar functions in *Hydra*.

### Defense molecules

**Figure 4:**
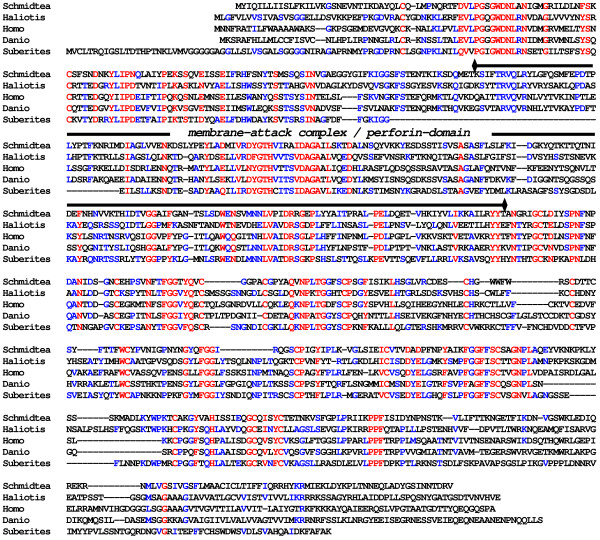
**A potential perforin exist in *Schmidtea *that is related to other known perforins**. We identified a perforin-like protein in the *Schmidtea *cDNA library. Using corresponding EST sequence and further EST sequences from the public database (DN313739, EG343971, DN299412, DN307006) we constructed the potential full-length protein. The alignment with perforin sequences from *Haliotis corrugata *(AAR82935), *Homo sapiens *(AAI12231), *Danio rerio *(XP_688214), and the sponge *Suberites domuncula *(CAI68018) reveal that perforins are conserved in evolution. Red color indicates 90% consensus and blue color 40% consensus.

In this study we describe for the first time a platyhelminth protein with a relationship to the mammalian perforin, the macrophage-expressed protein, (Fig. 4) that probably functions in the *Schmidtea *innate immune system. Similar proteins have also been identified in porifera and cnidaria [38,39] suggesting that perforins are evolutionarily conserved defense molecules. That also applies for an identified *Hydra *PR-1 (pathogenesis-related proteins-1) protein that shares sequence similarities with immune-inducible plant PR-1 proteins. The PR-1 family is strongly conserved in plants, fungi, insects, and vertebrates, including humans, and some recent studies suggest a role as signaling or effector molecule in antifungal defense reactions in plants [40].

**Figure 5:**
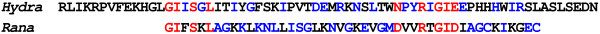
**A potential antimicrobial peptide in *Hydra***. We identified a *Hydra *cDNA (FC823225) that encodes a cationic peptide with predicted α-helical structure [67]. It shares significant similarities with antimicrobial esculentin-1B (P40844) from *Rana esculenta*.

Generation of reactive oxygen species by phagocytes is an essential mechanism of mammalian host defense against microbial infections. Several protein kinase C isoforms have been found to phosphorylate p47phox (neutrophil cytosolic factor 1), resulting in its membrane translocation and activation of the NADPH oxidase [41]. Here, we identified a *Hydra *protein with highest similarities to p47phox from other animals suggesting that generation of reactive oxygen species may also be important for antimicrobial defense in Cnidaria. In addition, we determined several short open reading frames in *Schmidtea *and *Hydra *that may encode potential antimicrobial peptides or signaling molecules. For example, one identified cDNA encodes a putative antimicrobial peptide that is cationic with potential α-helical structure. It shows significant similarities to antimicrobial esculentin-1B from the frog *Rana esculenta *(Fig. 5). This is in agreement with our observations that *H. vulgaris *homogenates display inducible antimicrobial activities as determined by the inhibition zone assay against live *Micrococcus luteus *bacteria (data not shown).

Interestingly, one immune-inducible transcript from *Hydra *exhibits significant sequence similarities to major vault proteins from other organisms. Very recently, major vault protein were found to be rapidly recruited to lipid rafts when human lung epithelial cells are infected with *Pseudomonas aeruginosa*. Major vault protein has been demonstrated to be essential for optimal epithelial cell internalization and clearance of *P. aeruginosa *indicating that it makes a substantial contribution to epithelial cell-mediated resistance to infection in mammals [42] and probably also in Cnidaria. However, the group of Thomas C.G. Bosch has recently described that in *Hydra*, similar as in humans, the immune system maintains a substantial resident beneficial microbiota on their epithelia [43]. This suggests that *Hydra *is able to discriminate 'friend' from 'foe' by killing entities that do damage and let those live that are commensals or mutualists which is in agreement to Poly Matzingers' proposed danger model of mammalian immunity [44] and our recent findings from insects that endogenous alarm signals induce innate immune responses during infection [45,46].

### Cellular homeostasis, cell adhesion related proteins, and regeneration

**Figure 6:**
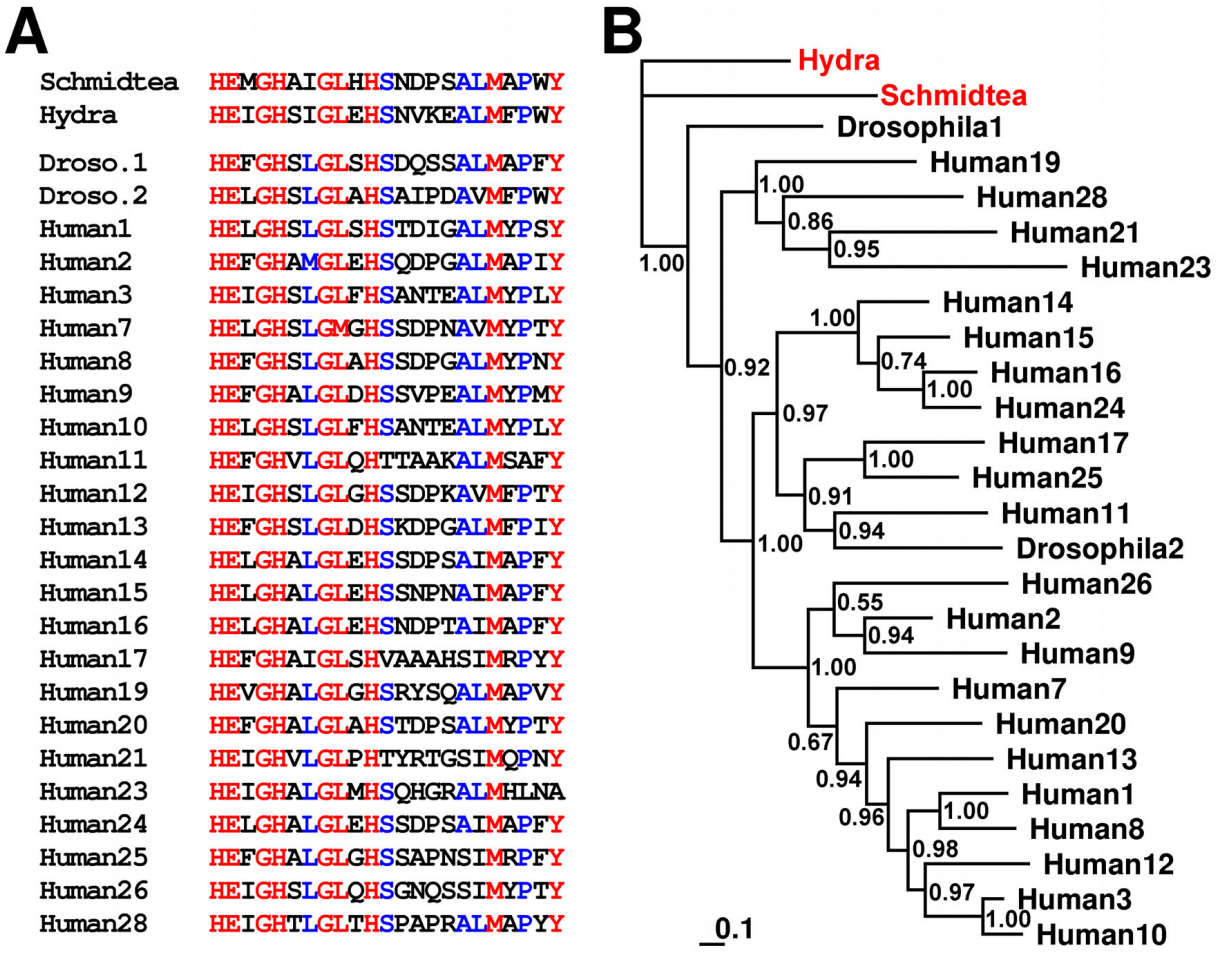
**An identified *Schmidtea *MMP gene is related to *Hydra *MMP and to MMPs from insects and mammals**. **(A) **All known MMPs from the fully genome sequenced ecdysozoan *Drosophila melanogaster *and humans (Deuterostomia) were aligned with the *Schmidtea*-MMP (AY068367) and the active site sequences are shown (MMP active site consensus sequence: HEXGHXXGXXHS). **(B) **A Bayesian protein tree was generated and we found that the *Schmidtea*-MMP grouped together with the MMP from *H. vulgaris *(AAD45804) nearest to MMP-1 from *D. melanogaster*. UniProt accession numbers for MMPs are: Drosophila1, Q9GTK3; Drosophila2, Q8MPP3; Human19, Q99542; Human28, Q9H239; Human11, P24347; Human21, Q8N119; Human17, Q9ULZ9; Human25, Q9NPA2; Human14, P50281; Human15, P51511; Human16, P51512; Human24, Q9Y5R2; Human20, O60882; Human12, P39900; Human13, P45452; Human1, P03956; Human8, P22894; Human3, P08254; Human10, P09238; Human7, P09237; Human26, Q9NRE1; Human23, O75900; Human2, P08253; Human9, P14780. Posterior probabilities are plotted at nodes. The scale bar represents the substitutions per site.

We identified several *Schmidtea *and *Hydra *proteins potentially involved in cellular homeostasis such as ribosomal proteins, myosin, actin, tubulin and metabolic proteins including *Schmidtea *isoprenoid biosynthesis enzyme 3-hydroxy-3-methyl-glutaryl-CoA reductase (HMG-CoA reductase) and *Hydra *glycolytic enzyme enolase. This may reflect the need of an increased cellular metabolism during tissue regeneration. In addition, we determined a *Hydra *dickkopf-like protein that is potentially involved as an antagonist in Wnt signaling [47]. Confirming our result, a recent study demonstrated that the *Hydra *dickkopf-like protein expression is stimulated by the injury signal itself [48]. In vertebrates, wound healing and formation of a specialized wound epidermis require Wnt/β-catenin signaling and, additionally, the action of matrix metalloproteinases (MMPs) [49]. In line with this, we identified a septic-injury inducible MMP homologue in *Schmidtea *that is evolutionarily conserved (Fig. 6). In *Hydra*, MMPs were shown to be required in extracellular matrix degradation and epithelial morphogenesis [50,51]. Members of this evolutionarily conserved family of enzymes play well-established multifaceted roles in tissue remodeling due to their capacity to degrade the extracellular matrix [52] and have recently been recognized as important modulators of immunity both in mammals [53] and in insects [54].

In addition, we found a *Hydra *protein with highest similarities to vWF (von Willebrand factor) proteins from other animals. This vWF protein is crucial for vertebrate blood clotting by binding to platelet receptors and collagens [55] and, in *Hydra*, may be important for cell-cell or cell-basement membrane adhesion processes.

**Figure 7:**
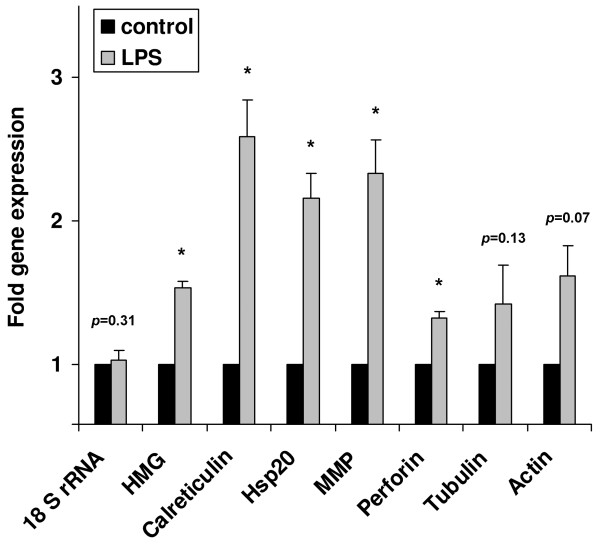
**Quantitative real time RT-PCR analysis of selected *Schmidtea *genes that are up-regulated in response to septic wounding**. The mRNA levels of selected genes in immune challenged animals (gray bars) were determined and are shown relative to their expression levels in untreated animals (black bars). The expression of the house-keeping gene 18S rRNA was not significantly influenced by the treatment. Results represent mean values of three independent determinations ± SD. Statistically significant differences were determined using Student's t-test and are indicated by asterisks (p < 0.05) or the determined *p *values were inserted.

Finally, we performed quantitative real time RT-PCR analyses using RNAs from untreated and immune challenged *Schmidtea *to precisely determine expression levels of several selected immune-inducible genes that were identified in the present study. This analysis confirmed the statictically significant induced expression of HMG-Co-A reductase, calreticulin, Hsp20, MMP, and perforin in response to septic injury (Fig. 7). The mRNA levels of tubulin and actin genes were elevated upon wounding but without statistically support due to higher variations of results from different determinations.

## Conclusion

Here, we report a comparative analysis of immune-inducible genes in two phylogenetically distant model organisms of regeneration and stem cell research. We selected the cnidarian *H. vulgaris *and the planarian *S. mediterranea *for targeted screening of genes that are up-regulated upon large-scale septic wounding using the SSH method. Obtained results highlight the ancient origin of some genes (e.g. MMPs, calreticulin, p47-phox, perforin, and major vault protein) known from vertebrate immunity. This is consistent, for instance, with the findings that even the most ancient animals, porifera, possess functional Toll-like receptors [56] and with the recent report of a bioinformatic analysis of the immune repertoire in Cnidaria [39]. However, screening for immunity-related genes in genomic or EST databases only allows identification of genes that share sequence similarities with known genes, while the SSH-approach is particularly suitable for the experimental screen for yet unknown or unexpected genes and, therefore, complement the bioinformatic approaches. Confirming this, the present study increases the number of genes identified in *Schmidtea *and *Hydra *(e.g. phospholipase C and perforin for *S. mediterranea *and major vault protein and vWF protein in *H. vulgaris*). In addition, using the SSH-method we have recently identified novel insect antimicrobial peptides among which some of them emerged as promising templates for the rational design of second generation antibiotics [57] or as transgenes for the generation of disease-resistant crops [58]. Hence, identified immunity-related genes from *Schmidtea *and *Hydra *may have also potential therapeutic value. Additionally, it will be a challenge to elucidate physiological functions of the presently identified genes during immune responses and homeostasis and to employ *Schmidtea *and *Hydra *as model organisms for the investigation of molecular interactions of pathogens with the host innate immune system.

## Methods

### Immune challenge of *Schmidtea mediterranea *and *Hydra vulgaris *and RNA isolation

The asexual strain of *S. mediterranea *(originated from a fountain in Montjuïc, Barcelona, Spain) was kept at 18°C in darkness and fed once per week with sheep liver. One-week-starved about 7-mm-long animals were used for experiments. *H. vulgaris *(Zürich strain, originally isolated by P. Tardent) was cultured at 18°C as described [59]. Septic wounding was performed by dissecting animals in two parts using a scalpel in the presence of 50 μg/ml LPS (purified *Escherichia coli *endotoxin 0111:B4, Cat. No.: L2630, Sigma, Taufkirchen, Germany). Total RNA was extracted from 14 h post immune challenged animals using the TriReagent isolation reagent (Molecular Research Centre, Cincinnati, Ohio, USA) according to the instructions of the manufacturer. RNA integrity was confirmed by ethidium bromide gel staining and quantities were determined spectrophotometrically.

### Construction of subtracted cDNA libraries using the SSH method

In order to identify genes that are differentially expressed in response to septic injury we performed the suppression subtractive hybridization method using RNAs from immune challenged and untreated *S. mediterranea *and *H. vulgaris*, respectively, the SMART PCR cDNA synthesis Kit (Clontech, Mountain View, CA, USA), and the PCR-Select cDNA subtraction Kit (Clontech), according to the protocols of the manufacturer. Colony PCR of each 288 randomly picked colonies and blot hybridization have been performed similar as described recently [19].

### Sequencing and computer analysis of cDNA sequence data

Plasmid isolation of positively screened colonies was performed with the FastPlasmid Mini Kit (Eppendorf, Hamburg, Germany) and purified plasmids were custom sequenced by Macrogen Inc. (Seoul, South-Korea). Blast [60] was used to identify corresponding gene sequences in the public sequence databases. InterProScan [61] was used for an integrated search in PROSITE, Pfam, and PRINTS databases at EMBL-European Bioinformatics Institute and to predict signal sequences and transmembrane regions.

### Sequence alignments and phylogenetic analysis

Multiple sequence alignments were computed using blosum62 program [62]. For phylogenetic reconstruction, we used the software package MrBayes 3.1.2 [63] which combines Bayesian inference and Markov chain Monte Carlo convergence acceleration techniques known as Metropolis coupling. The best fixed-rate model of amino acid evolution was determined by model jumping among nine possible models. The model with the overall highest posterior probability was WAG model [64] for the MMPs after 10^6 ^generations and for calreticulins after 2 × 10^6 ^generations. We used convergence diagnostic (i.e., the standard deviation of split frequencies) to determine whether the run length is sufficient. The average standard deviation of split frequencies was 0.0051 for MMPs and 0.0023 for the calreticulins. This indicated that the two chains that were run converged on similar results in all cases. The 50% majority rule tree presented here was constructed from all sampled trees with the first 25% of all trees ignored as burn in. Posterior probabilities plotted at the nodes can be interpreted as the probability that the tree or clade is correct [65].

### Quantitative real-time PCR

Quantitative real time RT-PCR was performed with the real-time PCR system Mx3000P (Stratagene, La Jolla, California, USA) using the FullVelocity SYBR^® ^Green QRT-PCR Master Mix (Stratagene), according to the protocols of the manufacturer. 50 pg of RNA per reaction were used to amplify 18S rRNA and 50 ng of RNA per reaction to amplify selected *Schmidtea*-genes using appropriate primers (Table 3). Primers were selected using the primer3 software [66] and were purchased from Thermo electron (Waltham, MA, USA).

**Table 3:** Genes analyzed by quantitative real time RT-PCR.

Gene	accession number	Forward primer 5'-3'	Reverse primer 5'-3'
18S rRNA	EG412600	ATGGTTGCAAAGCTGAAACT	TCCCGTGTTGAGTCAAATTA
HMG	EE666986	CGCATCTGATCCATGCAAGC	TGTACCTCCCCCGATTGTTCC
Calreticulin	EG417940	CCCTTGGGTGCATCCAGAAA	CGGACTTCACCTGCCACAGA
Hsp20	DN302574	GGAATCTGGGGTGAGCTTGG	CGGTTGATGGCTCAATGCAC
MMP	AY068367	TCGGCTTCTGGTTCCGATGT	CGGGCAAAAGCTGCAGAACT
Perforin	DN307006	GGCTGCCTGCATCTGTTTGA	TTGAGCCGTAGTCCGCCAAT
Tubulin	DN315207	TCCGGGTGGAGATTTGGCTA	GCACGCTTGGCATACATTAGGTC
Actin	EC386294	ATCCTGGCATTGCTGATCGT	TGGGGGAGCAACAATCTTGA

## Competing interests

The authors declare that they have no competing interests.

## Authors' contributions

BA designed and carried out the experiments, performed the analyses, and drafted parts of the manuscript. AV participated in its design and coordination, and drafted parts of the manuscript. All authors read and approved the final manuscript.
